# Sordarin, an antifungal agent with a unique mode of action

**DOI:** 10.3762/bjoc.4.31

**Published:** 2008-09-05

**Authors:** Huan Liang

**Affiliations:** 1 Department of Chemistry, The University of British Columbia, 2036 Main Mall, Vancouver, BC V6T 1Z1, Canada

**Keywords:** antifungal, bioactivity, sordaricin, sordarin, total synthesis

## Abstract

The sordarin family of compounds, characterized by a unique tetracyclic diterpene core including a norbornene system, inhibits protein synthesis in fungi by stabilizing the ribosome/EF2 complex. This mode of action is in contrast to typical antifungals, which target the cell membrane. This unusual bioactivity makes sordarin a promising candidate for the development of new fungicidal agents, and provided the motivation for extensive research. Three total syntheses (by the Kato, Mander and Narasaka groups), modifications of the glycosyl unit, and changes to the diterpene core (Cuevas and Ciufolini models) will also be discussed in this review.

## Introduction

**Figure 1:**
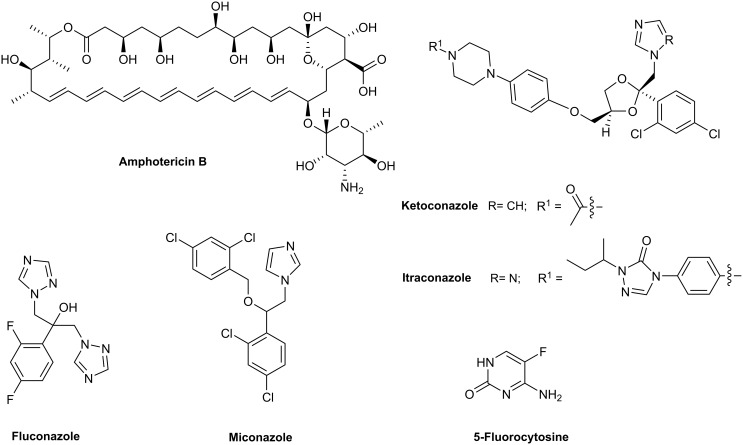
Therapeutic antifungal agents.

For immunosuppressed patients, especially those suffering from AIDS or cancer, fungal infections have become a significant, often life-threatening problem [1–2]. Present treatments rely on antifungals such as polyene antibiotics (amphotericin B), nucleoside analogs (5-fluorocytosine) and azoles (fluoconazole, itraconazole, ketoconazole, miconazole) (Figure 1). However, these drugs elicit severe side effects, for instance, nephrotoxicity; furthermore, they are ineffective against emerging resistant fungal strains. Therefore, the search for new antimycotic agents continues unabated. While the development of new therapeutic resources may entail the modification of existing agents, the identification of new antifungals that act on novel molecular targets is especially desirable. Sordarins fulfill this criterion.

**Figure 2:**
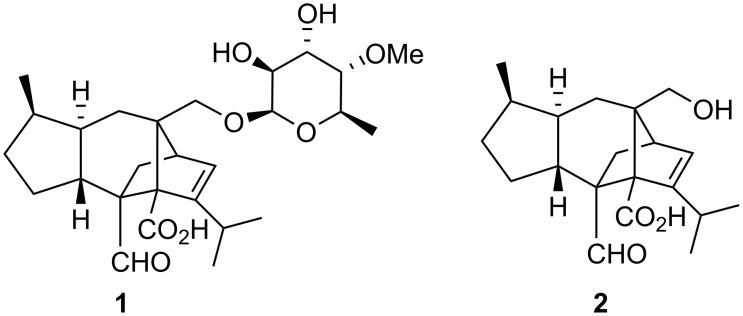
Structure of sordarin (**1**) and sordaricin (**2**).

Sordarin (**1**) was isolated in 1969 from the fungus *Sordaria araneosa* by scientists at the Sandoz Co., in Switzerland, and it was patented as SL 2266 [3–4]. The degradation of sordarin with concentrated aqueous HCl in acetone released a diterpenoid aglycone called sordaricin (**2**) (Figure 2). Sordarin production by fermentation was optimized to simplify purification [5] and to increase the yield [6]. Interestingly, early antifungal screens in the 1970s excluded sordarin, but two decades later, renewed appreciation of that natural product arose as a consequence of its potent *in vitro* inhibition of protein synthesis in *Candida albicans*, a pathogenic fungus.

Unlike traditional antifungal agents, which target only the integrity of the cell membrane through binding of ergosterol or inhibition of its biosynthesis [2,7], sordarin acts on elongation factor 2 (EF2). This enzyme catalyzes the translocation of the ribosome along mRNA during elongation of the emerging polypeptide chain [8]. Sordarin inhibits this translocation by stabilizing the EF2/ribosome complex. Strong activity against *Saccharomyces cerevisiae* [9–10], *Candida albicans* [11–12], and a number of pathogenic fungi make sordarin a promising antimycotic agent.

## Review

### Total syntheses of sordarin and its congeners

To date, three total syntheses of sordarin and its congeners have been published. The first syntheses of sordaricin methyl ester and its Δ^2^-derivative were achieved by Kato in 1993 [13]. Then the Mander group completed sordaricin in 2003 [14–15] and the Narasaka group reported total syntheses of racemic sordaricin [16] and enantiopure sordarin [17] in 2004 and 2006, respectively. Both Kato’s and Mander’s syntheses employed intramolecular Diels-Alder cyclizations to construct the norbornene-like framework, while an intramolecular Pd catalyzed Tsuji-Trost reaction was utilized by the Narasaka group to build the diterpene core.

### Kato’s synthesis of sordaricin methyl ester

**Scheme 1:**
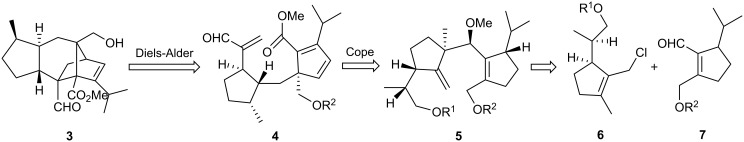
Kato’s retrosynthetic plan.

The first total synthesis of optically pure sordaricin methyl ester (**3**) was achieved by the Kato group in 1993, almost 24 years after the isolation of the natural product [13]. It relied on two key transformations for the construction of the tetracyclic diterpene core: a Cope rearrangement and an intramolecular Diels-Alder reaction. The substrate for the Cope rearrangement was prepared by the merger of two cyclopentene units (Scheme 1).

**Scheme 2:**
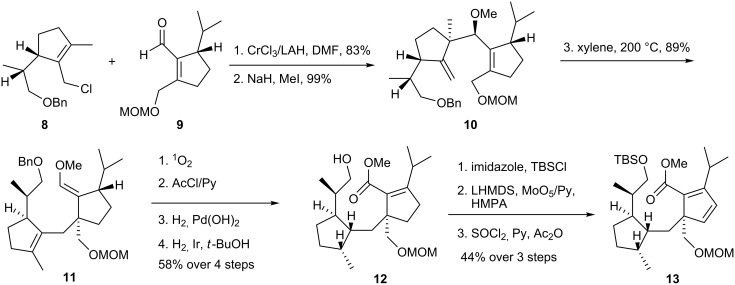
Synthesis of cyclopentadiene **13**.

First, the addition of chloro compound **8** [18] to aldehyde **9** [19] occurred smoothly using the CrCl_3_/LAH system, developed by Hiyama et al. [20]. The resultant alcohol was converted to the methyl ether using NaH and MeI. The subsequent Cope rearrangement of **10** occurred at 200 °C via a boat-like transition state [18] to yield **11**. The elaboration of this material to **12** involved treatment with singlet oxygen and AcCl [21] to yield α,β-unsaturated ester, which was subjected to Pearlman hydrogenolysis of the benzyl group and selective catalytic reduction of the non-conjugated olefin with iridium black. The latter step proceeded in a predominately *syn* manner to afford intermediate **12**, which was oxidized to cyclopentadiene **13** through TBS protection of the alcohol, MoO_5_-mediated hydroxylation of the enolate of the ester [22] and ensuing dehydration by SOCl_2_ (Scheme 2).

**Scheme 3:**
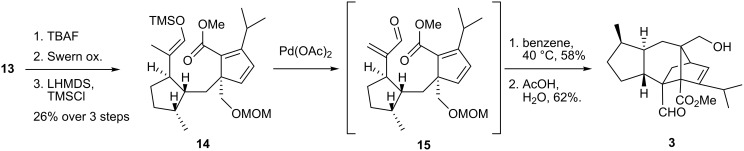
Synthesis of sordaricin methyl ester.

The sequence utilized for the introduction of the dienophilic subunit in **13** is outlined in Scheme 3. The primary alcohol was deprotected (TBAF) and oxidized to an aldehyde (Swern), which was converted to the corresponding silyl enol ether. Saegusa oxidation [23] of the latter occurred regioselectively to afford **15**, which underwent intramolecular cycloaddtion in benzene at 40 °C. A final deprotection gave sordaricin methyl ester (**3**) in 16 linear steps from **8** and **9** with an overall yield of 2%.

### Mander’s synthesis of sordaricin

**Scheme 4:**
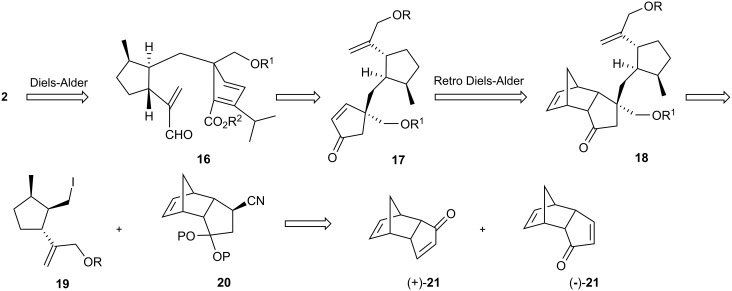
Mander’s retrosynthetic plan.

As early as 1991, Mander reported model studies [24–25] for sordaricin synthesis using intramolecular [4+2] cycloaddition, and this work culminated in a total synthesis of Sordaricin in 2003 [14–15]. Scheme 4 depicts Mander’s retrosynthetic analysis, which envisions the preparation of fragments **19** and **20** from the optical isomers of oxodicyclopentadiene **21** [26–27]. Thus, iodide **19** prepared from compound (+)-**21** would be coupled with nitrile **20**, obtained from (−)-**21**. Sordaricin (**2**) would be completed by retro- and normal Diels-Alder reactions.

**Scheme 5:**
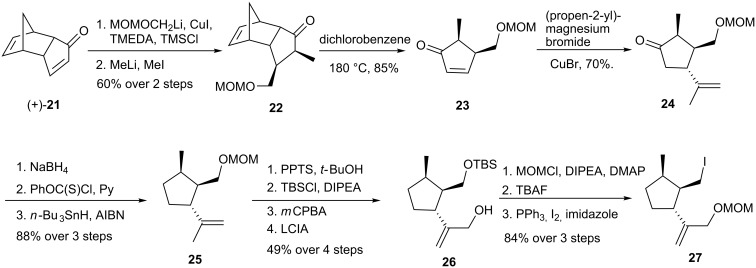
Synthesis of iodo compound **27**.

The synthesis of compound **22** began with double alkylation of (+)-**21** through a cuprate conjugate addition followed by treatment of the resulting enolate with methyl iodide (Scheme 5). Retro-Diels-Alder reaction of **22** in refluxing 1,2-dichlorobenzene gave α,β-unsaturated ketone **23**. Trisubstituted cyclopentanone **25** was obtained through another cuprate addition, followed by NaBH_4_ reduction of the ketone and subsequent deoxygenation under Barton-McCombie conditions.

**Scheme 6:**
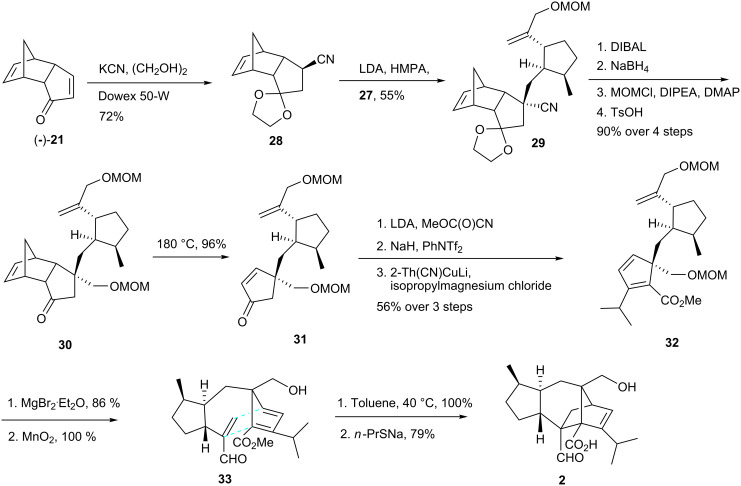
Synthesis of sordaricin (**2**).

Exchange of the MOM for a TBS protecting group and treatment with *m*CPBA furnished an epoxide, which was converted into allylic alcohol **26** by reaction with lithium cyclohexyl(isopropyl)amide (LCIA) in 49% overall yield for the 4-step sequence. The elaboration of intermediate **27** entailed a protection-deprotection maneuver that prepared the molecule for an ultimate Appel reaction [28] as a way to install the iodo substituent. The second key intermediate **29** was obtained in two steps starting from compound (−)-**21** (Scheme 6), *via* 1,4-addition of potassium cyanide followed by protection of the ketone as the ethylene ketal. Alkylation of the carbanion of nitrile **28** with iodide **27** provided exclusively diastereoisomer **29**.

Stepwise reduction (DIBAL and NaBH_4_) of the nitrile, MOM protection of the newly formed primary alcohol, and hydrolysis of the dioxolane gave ketone **30** and occurred as a prelude to retro-Diels-Alder reaction of the latter, leading to the α,β-unsaturated ketone **31**. Compound **32** was obtained by treatment with LDA and Mander’s reagent, formation of an enol triflate using phenyl triflimide and installation of the isopropyl group by an addition-elimination sequence using a thienylcuprate reagent. Removal of the MOM groups and selective oxidation of the allylic alcohol by MnO_2_ led to the cycloaddition substrate **33**, heating of which at 40 °C resulted in quantitative formation of sordaricin methyl ester. Nucleophilic cleavage of the methyl ester by *n*-PrSNa led to sordaricin (**2**). In short, a convergent synthesis of sordaricin was accomplished in 27 steps and in 3% overall yield from (+)- and (−)-oxodicyclopentadiene (**21**).

### Narasaka’s synthesis of sordarin and sordaricin

**Scheme 7:**
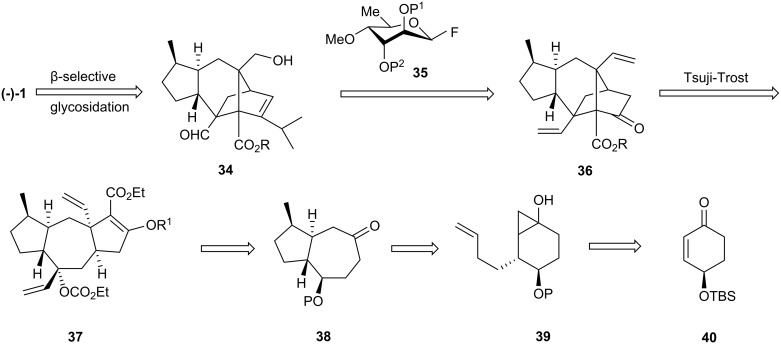
Retrosynthesis of sordarin and sordaricin.

Narasaka et al. published a synthesis of racemic sordaricin in 2004 [16], and 2 years later, they described the first enantioselective synthesis of (−)-sordarin [17]. The retrosynthetic plan is outlined in Scheme 7. (−)-Sordarin would result through a β-selective glycosylation reaction of sordaricin ester **34** with glycosyl fluoride **35**. Unlike previous syntheses, the present one utilized an intramolecular Tsuji-Trost reaction [29] of allylic carbonate **37** to build the core of sordaricin **36**.

In turn, compound **37** was prepared from bicyclic ketone **38**, which was derived from enantiopure 4-silyloxycyclohexenone **40** via bicyclo[4.1.0]heptanol **39**.

**Scheme 8:**
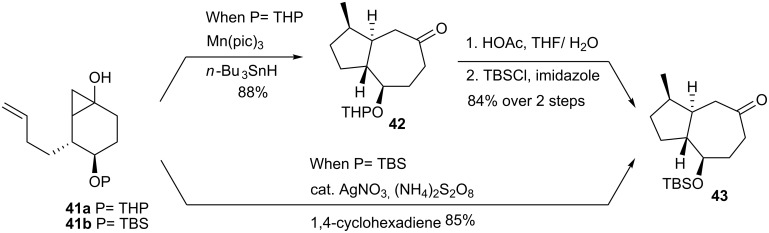
Synthesis of ketone **43**.

The synthesis of *rac*-sordaricin evolved from bicyclic ketone **43**, which resulted from treatment of (±)-**41a**, with a stoichoimetric amount of Mn(III) tris(pyridine-2-carboxylate) [30–31] (Scheme 8). The mechanism of conversion of **41a** to **42** is believed to involve single-electron oxidation of the cyclopropanol, fragmentation of a transient radical cation, and diastereoselective radical cyclization. In the enantioselective synthesis, this transformation was more efficiently achieved by treatment of enantioenriched **41b** with the AgNO_3_-ammonium persulfate-pyridine system [32]. The ensuing Ag(I) catalyzed oxidative radical cyclization proceeded in 85% yield.

**Scheme 9:**
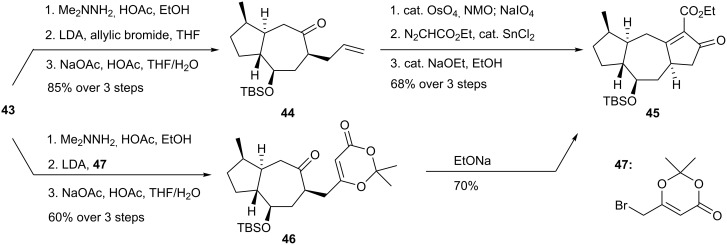
Synthesis of β-keto ethyl ester **45**.

Racemic ketone **43** underwent regio- and stereoselective allylation under Corey-Enders conditions to provide olefin **44**, which was subjected to Lemieux-Johnson cleavage to corresponding aldehyde (Scheme 9). The latter was directly converted to a β-ketoester by reaction with ethyl diazoacetate in the presence of SnCl_2_. A final Knoevenagel cyclization afforded substance **45**. The enantioselective synthesis implemented an improved procedure that relied on alkylation of the metallohydrazone of **43** with dioxenone **47**. This allowed the Knoevenagel condensation to be performed directly.

**Scheme 10:**
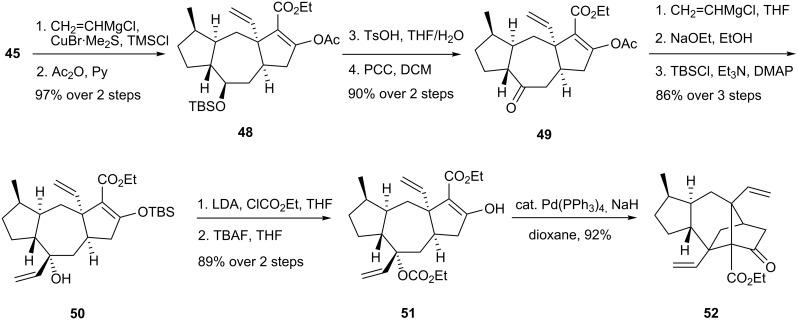
Synthesis of tetracyclic framework **52**.

In both the racemic and asymmetric syntheses, tricyclic compound **48** was prepared by Cu(I)-catalyzed conjugate addition of vinylmagnesium chloride to **45** in the presence of TMSCl, followed by enol acetylation (Scheme 10). Selective cleavage of the TBS group and PCC oxidation surrendered ketone **49**. Diastereoselective addition of vinylmagnesium chloride and exchange of the enol protecting group gave allylic alcohol **50**. The substrate **51** for the Tsuji-Trost reaction was prepared by carbethoxylation of the allylic alcohol and release of the TBS group. The advanced tetracyclic intermediate **52** emerged in 92% yield upon exposure of **51** to NaH in the presence of catalytic Pd(PPh_3_)_4_. The structure of **52** was verified by X-ray analysis.

**Scheme 11:**
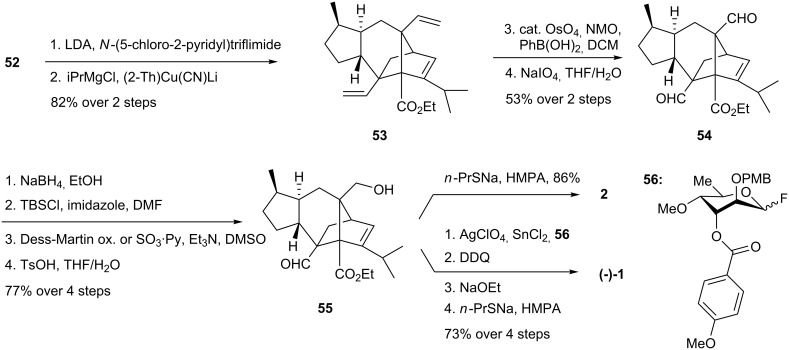
Synthesis of sordaricin and sordarin.

The isopropyl group was installed by enol triflation of the ketone using the Comins reagent, followed by displacement of the vinylic triflate using a higher order cuprate obtained from isopropylmagnesium chloride. Several alternatives were explored to introduce the isopropyl group; for instance, using Pd(0) or CeCl_3_ induced coupling reactions. However, none were successful, seemingly due to steric and electronic constraints (Scheme 11).

Selective Lemieux-Johnson cleavage of the vinyl groups using OsO_4_ and NaIO_4_ in the presence of PhB(OH)_2_ [33] afforded the dialdehyde **54**. Racemic sordaricin (**2**) was completed after a few standard redox and protective group manipulations. The synthesis of enantioenriched sordaricin proceeded in a like fashion. Finally, (−)-sordarin was reached starting with glycosidation of sordaricin ethyl ester **55** with fluoride **56**. This step relied on a Mukaiyama diastereoselective glycosidation [34], wherein the *p*-methoxybenzoyl group provides 1,3-anchimeric assistance during the departure of the anomeric fluoride, ultimately securing the β-configuration of newly formed glycosyl bond. Full deblocking of the product of this reaction required 3 steps and provided (−)-sordarin in an overall reported yield of 5% from compound **41** over 27 steps.

## Synthetic analogs of sordarin

### Glycosyl analogs

**Figure 3:**
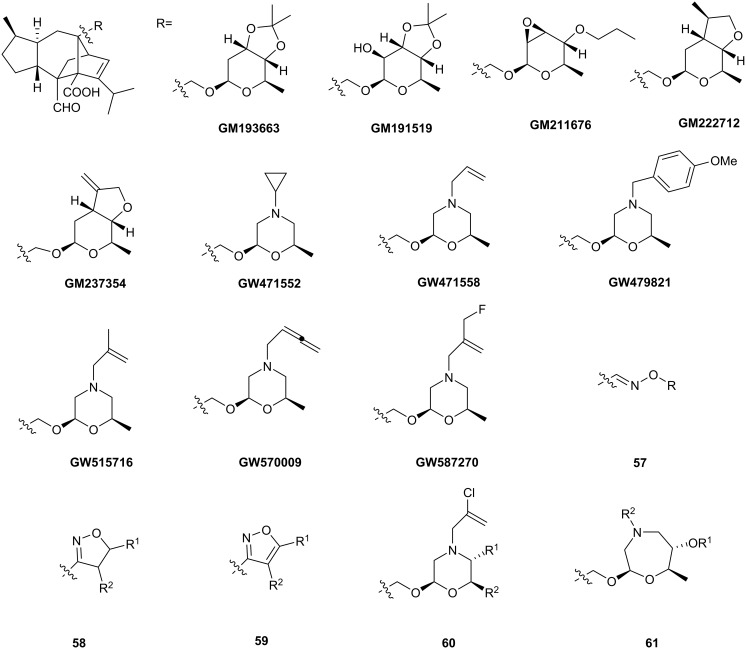
Modifications of glycosyl part.

Sordarin and congeners exhibit good activity against some fungal species, for instance, *Candida albicans* [11–12], but are inactive toward others. Modifications to both the glycosyl unit and the diterpene core have been pursued in an effort to engender activity against the greatest possible number of pathogenic fungi. Leading pharmaceutical laboratories such as Merck [35], GSK [36–37], Bristol-Myers-Squibb [38–39] and Sankyo [40–41], have focused on modification of the carbohydrate moiety. Several representative analogs from the so-called GM series developed by GSK in 1998 are shown in Figure 3. Of note is their activity against a variety of human pathogens belonging to the *Candida*, *Aspergillus*, and *Pneumocystis spp*. In 2001, the same company released the synthesis of a new series of “GW” analogs named “azasordarins”. Similar to superior activity was observed against the abovementioned pathogens with respect to the first GM generation. Toxicity studies on mammalian (including human) cell lines revealed a lower *in vitro* toxicity than the clinically used compound, amphotericin B.

Oxime **57**, isoxazoline **58** and isoxazole **59** derivatives were prepared in the Bristol-Myers Squibb laboratories. They were generally less active than the GSK compounds. Yet another modification involving the introduction of oxazepane **61** functionalities was reported by Sankyo Co. The best results, obtained by substitution at the nitrogen position, still did not match the GM and GW series. Worth mentioning is the lack of extensive toxicity tests from the latter two companies.

### Diterpene skeleton analogs

Structure-activity relationship studies of sordarin have been largely confined to the glycosyl moiety, while the exact roles of the different components of the aglycone, such as the isopropyl group, the carboxylic acid, and the tetracyclic framework, are unknown. The total syntheses presented previously did not address these issues. However, it is established that the aldehyde function may be replaced by a nitrile group without loss of activity [42]. Efforts aiming to elucidate the role – if any – of the subunits of the aglycone have been described by Cuevas and by Ciufolini. Highlights of these contributions are outlined below.

### Cuevas’s cyclopentane analog

**Scheme 12:**
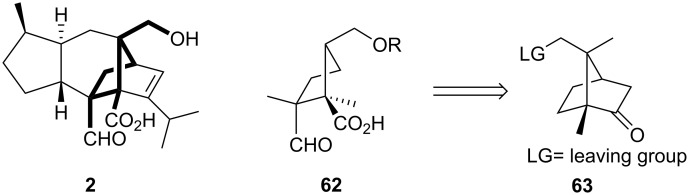
Simplified model of sordarin.

Modeling studies by Cuevas et al. relying on the INSIGHT software [43–44] reduced the tetracyclic framework of sordarin to a simple cyclopentane derivative **62** (Scheme 12), which contains aldehyde, carboxylic acid and hydroxymethyl groups as pharmacophores. The dihedral angles of CHO-CH_2_-CH_2_-COOH in the cyclopentane analog are very close to those of the carbon skeleton in sordaricin.

**Scheme 13:**
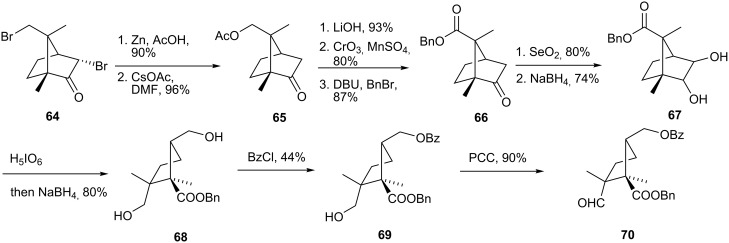
Synthesis of cyclopentane analog precursors.

The synthesis of **62** started with commercially available (+)-3,9-dibromocamphor **64**, which was advanced to acetoxycamphor by monodebromination by Zn/HOAc and substitution of the surviving halogen with CsOAc (Scheme 13). Substance **66** was reached by hydrolysis of the acetate, oxidation of the corresponding alcohol directly to the carboxylic acid and benzyl ester formation. Riley oxidation [45] of the ketone followed by reduction with NaBH_4_, afforded dihydroxy camphor derivative **67**. Periodic acid cleavage of the resulting vicinal diol and NaBH_4_ reduction *in situ* gave diol **68**. Selective benzoylation of the less hindered alcohol and PCC oxidation led to compound **70**.

**Scheme 14:**
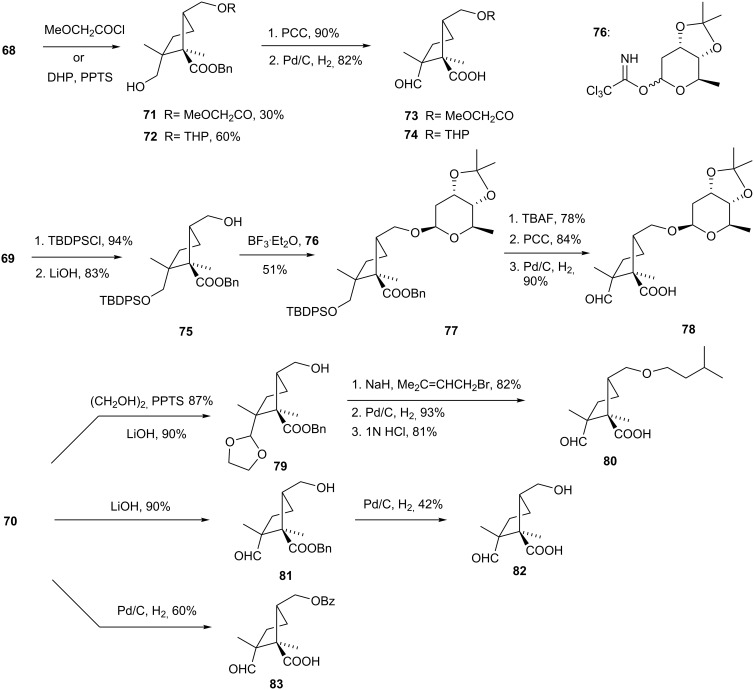
Synthesis of six cyclopentane analogs.

Six derivatives of **68**, **69** and **70** displaying varying lipophilicities were synthesized as indicated in Scheme 14. Thus, **68** was advanced to **71** and **72** by treatment with methoxyacetyl chloride or DHP and PPTS, respectively. PCC oxidation and Pd/C hydrogenation of **71** and **72** led to analogs **73** and **74**. Compound **69** converted into **75** by protection of the OH group with TBDPSCl and saponification of the benzoate ester (LiOH). Lewis acid-induced glycosylation [46] of **75** with trichloroacetimidate **76** gave **77**. The latter was then subjected to TBAF deprotection, PCC oxidation, and Pd/C hydrogenation, to afford analog **78**.

Isopentyl ether **80** was prepared via ethylene glycol protection, saponification, alkylation by prenyl bromide, hydrogenation and acetonide cleavage. Two other analogs were assembled from **70** by LiOH hydrolysis of the benzoate ester and Pd/C hydrogenolysis of the benzyl one. While biological tests indicated that compared to sordaricin (**2**) the cyclopentane analogs showed generally reduced potency towards a *C. albicans* protein synthesis assay, as well as diminished suppression of *C. albicans* cell growth, some analogs were actually more potent in certain tests. For instance, analogs **74**, **78**, **83** were all more potent than sordaricin in the *C. albicans* 2005E assay, showing between 3.6 and 6.7 times lower MICs compared to sordaricin. These promising results indicate that simplification of the sordarin skeleton is feasible.

### Ciufolini’s tricyclic analogs

**Scheme 15:**
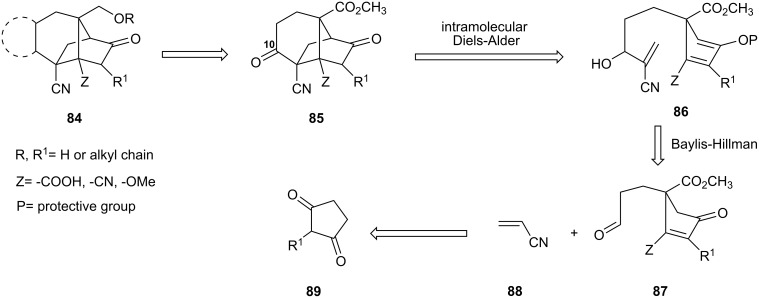
Retrosynthetic plan of sordarin analog.

The search for improved potency led us to explore further skeletal modifications. The goal of this study, which was carried out in collaboration with the Bayer CropScience Co., was to develop a general access to analogs of sordarin (cf. **84** in Scheme 15) with a focus on simplification of its synthesis [47]. The approach relies on an intramolecular Diels-Alder reaction leading to a series of tricyclic analogs **85** displaying various functional groups. The C-10 ketone in this material would enable annulation sequences leading to tetracyclic compounds **84**. The precursor to **85**, intermediate **86**, would arise from a Baylis-Hillman reaction [48] between aldehyde **87** and acrylonitrile (**88**), obtained in turn from 2-alkylcyclopentanedione **89**.

**Scheme 16:**
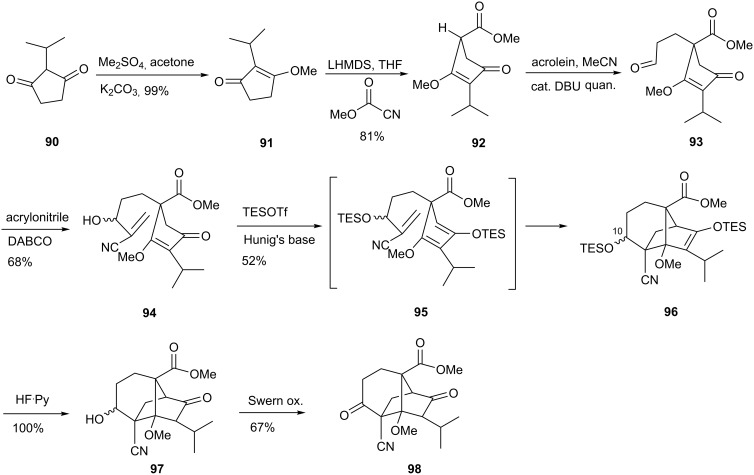
Synthesis of sordarin analog **98**.

Diketone **90** was advanced to vinylogous ester **91**, the enolate of which was acylated with the Mander reagent to furnish **92** (Scheme 16). The subsequent Michael addition of **92** to acrolein proceeded in quantitative yield under the catalytic influence of DBU. This reaction worked best when DBU was added to a mixture of ester **92** and acrolein in acetonitrile. Much poorer results were obtained by operating in THF. Moreover, purification of the aldehyde was found to be troublesome due to its instability on silica. Fortunately, reactions carried out in MeCN afforded a product of excellent quality that was directly utilized in the next step. Then, treatment of crude **93** in acrylonitrile with a catalytic amount of DABCO gave the Baylis-Hillman product **94**. Preparation of the Danishefsky-like diene **95** by treatment of **94** with Hunig’s base and TES triflate triggered a simultaneous intramolecular Diels-Alder reaction that produced **96** in 52% yield. The compound was obtained as a 67:33 mixture of unassigned diastereomers of the C-10 TESO- group. Both TIPSOTf and TESOTf were effective in this step. However, later removal of the TIPS group either with TBAF or HF/pyridine complex was problematic. The problem was circumvented by using the less hindered TES group. Treatment of both diastereoisomers of **96** with HF/Pyridine complex led to a mixture of diastereoisomeric alcohols **97**. Swern oxidation of corresponding alcohols afforded the tricyclic sordaricin analog **98**. In summary, diketone **98** was obtained from starting compound **90** in an overall yield of 19% over 7 steps.

**Scheme 17:**
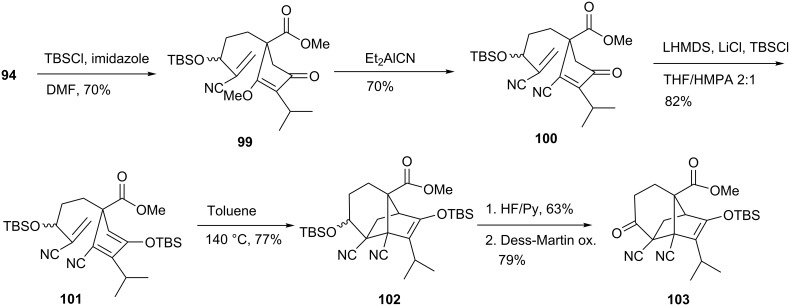
Synthesis of sordarin analog **103**.

The sordarin analogs shown in Scheme 16 exhibit a bridgehead methoxy instead of a carboxy group. Efforts to adapt the general strategy delineated above to the preparation of bridgehead carboxy derivatives more closely resembling **1** centered on the preparation of nitrile analog **103** (Scheme 17). It was anticipated that the cyano group could be easily introduced by 1,4-addition of cyanide ion to **99** and later converted into a carboxylic acid at an appropriate time.

To this end, treatment of substrate **94** with TBSCl in the presence of imidazole and a catalytic amount of DMAP provided **99**. The Nagata reagent [49] readily transformed **99** into **100**, presumably by 1,4-addition of cyanide ion and ensuing elimination of methanol. Unexpectedly, enone **100** was immune to the one-pot enol silylation / cycloaddition conditions that had successfully advanced **94** to **95**, and it was recovered unchanged after many such attempts. Thus, extensive studies were performed to find suitable conditions for the silylation reaction. Silyl enol ether formation failed to occur under Corey-Gross (LDA, R_3_SiCl) [50], Miller [(TMS)_2_NH, TMSI] [51], and Lewis acid-induced (ZnCl_2_, TiCl_4_) [52–53] conditions. The ketone also resisted the action of specialized reagents such as *N*,*O*-bis(trimethylsilyl)acetamide and *N*,*O*-bis(trimethylsilyl)trifluoroacetamide [54–55]. Ultimately, the desired compound **101** was obtained in 82% yield through an unusual procedure that involved reaction of **100** with a large excess of LHMDS and LiCl [56] in THF-HMPA in the presence of TBSCl. However, this material displayed no proclivity to undergo intramolecular Diels-Alder reaction at or near room temperature. Indeed, cyclization to **102** occurred only upon heating in anhydrous toluene at 140 °C (sealed tube). Fortunately, the reaction proceeded smoothly. Following HF deprotection and Dess-Martin oxidation, ketone **103** was obtained in an overall yield of 8% over 10 steps. Further biological studies will be performed to investigate the biological activity of **98**, **103** and analogs.

## Conclusion

The advent of practical synthetic avenues to the core of sordarin and its analogs should permit a thorough medicinal chemistry investigation of the role of individual components of the aglycone. Coupled with the observations recorded during efforts centering on modification of the glycosyl sector, this knowledge may well lead to new antifungal agents with a broad spectrum and potent activity.
